# Sternum wound contraction and distension during negative pressure wound therapy when using a rigid disc to prevent heart and lung rupture

**DOI:** 10.1186/1749-8090-6-42

**Published:** 2011-03-30

**Authors:** Sandra Lindstedt, Richard Ingemansson, Malin Malmsjö

**Affiliations:** 1 Department of Cardiothoracic Surgery, Lund University and Skåne University Hospital, Lund, Sweden; 2 Department of Ophthalmology, Lund University and Skåne University Hospital, Lund, Sweden

## Abstract

**Background:**

There are increasing reports of deaths and serious complications associated with the use of negative pressure wound therapy (NPWT), of which right ventricular heart rupture is the most devastating. The use of a rigid barrier has been suggested to offer protection against this lethal complication by preventing the heart from being drawn up against the sharp edges of the sternum. The aim of the present study was to determine whether a rigid barrier can be safely inserted over the heart with regard to the sternum wound edge movement.

**Methods:**

Sternotomy wounds were created in eight pigs. The wounds were treated with NPWT at -40, -70, -120 and -170 mmHg in the presence and absence of a rigid barrier between the heart and the edges of the sternum. Wound contraction upon NPWT application, and wound distension under mechanical traction to draw apart the edges of the sternotomy were evaluated.

**Results:**

Wound contraction resulting from NPWT was similar with and without the rigid barrier. When mechanical traction was applied to a NPWT treated sternum wound, the sternal edges were pulled apart. Wound distension upon traction was similar in the presence and absence of a the rigid barrier during NPWT.

**Conclusions:**

A rigid barrier can safely be inserted between the heart and the edges of the sternum to protect the heart and lungs from rupture during NPWT. The sternum wound edge is stabilized equally well with as without the rigid barrier during NPWT.

## Introduction

The use of negative pressure wound therapy (NPWT) for the treatment of deep sternal wound infections has been shown to have remarkable effects on healing [1]. There are, however, increasing numbers of reports of deaths and serious complications associated with the use of NPWT due to heart rupture, lung rupture, bypass graft bleeding and death; the incidence being 4 to 7% of all patients treated for poststernotomy mediastinitis with NPWT after cardiac surgery [2-4]. In November 2009, the FDA filed an alert, and the importance of protecting exposed organs during NPWT and this issue has also been emphasized in the international scientific literature [5-8].

We have previously elucidated the cause of heart rupture in pigs using magnetic resonance imaging [9,10]. The heart was shown to be drawn up towards the thoracic wall, the right ventricle bulged into the space between the sternal edges, and the sharp edges of the sternum protruded into the anterior surface of the heart, in some cases resulting in damage to the left ventricle of the heart or damage to a bypass graft to the right coronary artery [10]. Multiple layers of paraffin gauze over the anterior portion of the heart did not prevent the heart from being deformed. These events could, however, be prevented by inserting a rigid plastic disc between the anterior part of the heart and the inside of the thoracic wall [10]. Heart and lung ruptures similar to those seen in patients were observed in this experimental set-up without the rigid discs, while no damage to the heart or lungs was seen when the discs were used [10].

Several important aspects must be taken into consideration when treating a sternotomy wound with NPWT. The edges of the sternum move when the patient breaths, coughs and moves. Therefore, the sternum wound must be contracted and stabilized in order to allow adequate respiration and mobilization [5,11]. The aim of the present study was to investigate sternum wound contraction and distension in the presence and absence of a rigid barrier, inserted between the heart and the edges of the sternum, to protect the heart and lungs from damage and rupture during NPWT. Wound contractions were measured before and after negative pressures ranging from -40 to -170 mmHg were applied. Sternum wound distension during mechanical traction to pull apart the edges of the sternotomy, was evaluated using forces up to 320 N.

## Material and methods

### Animals

A porcine sternotomy wound model was used. Eight domestic landrace pigs with a mean weight of 70 kg were fasted overnight with free access to water. The study was approved by the Ethics Committee for Animal Research, Lund University, Sweden. The investigation complied with the "Guide for the Care and Use of Laboratory Animals" as recommended by the U.S. National Institutes of Health, and published by the National Academies Press (1996).

### Anaesthesia and surgery

Premedication was performed with an intramuscular injection of xylazine (Rompun^® ^vet. 20 mg/ml; Bayer AG, Leverkusen, Germany; 2 mg/kg) mixed with ketamine (Ketaminol^® ^vet. 100 mg/ml; Farmaceutici Gellini S.p.A., Aprilia, Italy; 20 mg/kg). Before surgery, a tracheotomy was performed and an endo-tracheal tube was inserted. Anaesthesia was maintained with a continuous infusion of ketamine (Ketaminol^® ^vet. 50 mg/ml; 0.4-0.6 mg/kg/h). Complete neuromuscular blockade was achieved with a continuous infusion of pancuronium bromide (Pavulon; N.V. Organon, Oss, the Netherlands; 0.3-0.5 mg/kg/h). Fluid loss was compensated for by continuous infusion of Ringer's acetate at a rate of 300 ml/kg/h. Mechanical ventilation was established with a Siemens-Elema ventilator (Servo Ventilator 300, Siemens, Solna, Sweden) in the volume-controlled mode (65% nitrous oxide, 35% oxygen). Ventilatory settings were identical for all animals (respiratory rate: 15 breaths/min; minute ventilation: 8 l/min). A positive end-expiratory pressure of 5 cmH_2_O was applied. A Foley catheter was inserted into the urinary bladder through a suprapubic cystostomy. Upon completion of the experiments, the animals were given a lethal dose (60 mmol) of intravenous potassium chloride.

### Wound preparation

**Figure 1:**
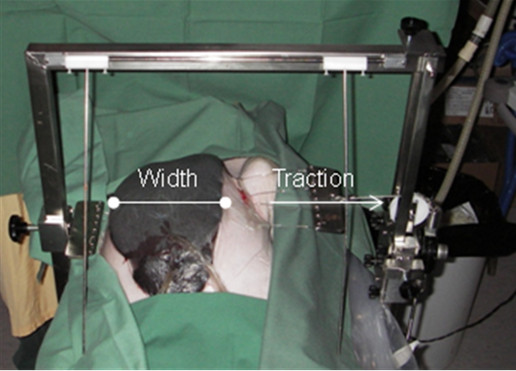
**Photograph of the experimental set-up used to measure wound distension upon the application of a lateral force during NPWT**. Negative pressure was applied with or without a rigid disc placed between the heart and the edges of the sternum. Two 6-0 steel wires were secured around the ribs on each side of the sternum and attached to a custom-made traction device. The traction device was connected to a force transducer and a recorder. Negative pressures of 0, -40, -70, -120 and -170 mmHg were applied. The wound width was measured when traction forces between 0 and 320 N were applied to the lateral edges of the sternotomy.

A midline sternotomy was performed and the pericardium and the pleurae were opened. Two 6-0 steel wires for use in sternal closure (Syneture, Tyco Healthcare, CT, USA) were secured around the ribs on each side of the sternum, and attached to a custom-made sternal traction device. The purpose of this was to test sternum wound distension when lateral traction was applied to draw apart the edges of the sternotomy (Figure 1). The traction device was connected to a force transducer and a recorder. The wound was treated with NPWT in the presence or absence of a rigid plastic disc, which was inserted between the heart and the sternum. The wound was filled with open-pore polyurethane foam. One layer of foam was placed between the sternal edges. A second layer of foam was placed over the first layer, between the soft tissue wound edges, and secured to the surrounding skin. The wound was sealed with a transparent adhesive drape, and the drain was connected to the vacuum source. The vacuum source was set to deliver negative pressures of -40, -70, -120 or -170 mmHg. The different negative pressures were applied in random order.

### Wound contraction

The distance between the lateral wound edges was measured. Measurements were performed before and after the application of negative pressures of -40, -70, -120 and -170 mmHg.

### Wound distension

Lateral traction was applied to the sternotomy wound, using the traction device described above, and the distension of the wound was measured. The effects of lateral forces, ranging from 0 to 320 N, were studied on the NPWT treated sternotomy wound, at the negative pressures of -40, -70, -120 and -170 mmHg. This was done to ensure that the sternum is sufficiently stabilized during NPWT to withstand the forces to which the wound is exposed when the patient breathes, coughs or moves.

### The protective disc

The protective disc was made out of bio-compatible plastic that could withstand a force of a negative pressure of at least -50 mmHg. The disc was 20 × 8 cm and was then cut to appropriate size to fit between the anterior part of the heart and the posterior part of the sternum. The disc had multiple small perforations all over the disc area to allow drainage. The disc was ridged with flexible edges.

### Calculations and statistics

Calculations were performed using GraphPad 5.0 software (San Diego, CA, USA). Statistical analysis was performed using the Mann-Whitney test when comparing two groups and the Kruskal-Wallis test with Dunn's post-test for multiple comparisons when comparing three groups or more. Significance was defined as p < 0.05. Results are presented as the mean of 8 measurements ± the standard error of the mean (S.E.M.).

## Results

### Wound contraction under NPWT

**Figure 2:**
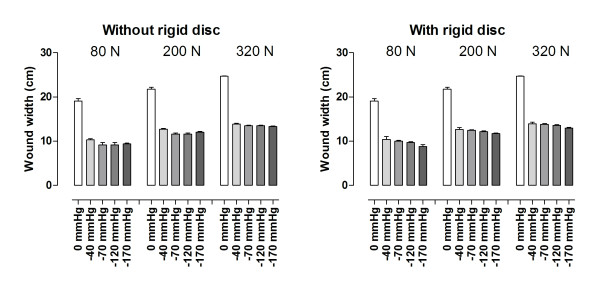
**Sternotomy wound contraction upon application of NPWT (-40, -70, -120 and -170 mmHg) with and without a rigid disc between the heart and the sternum**. Results are presented as mean values of 8 measurements ± S.E.M. It can be seen that the degree of wound contraction is similar in both settings.

Various negative pressures (-40, -70, -120 and -160 mmHg) were applied to the sternal wound and the width of the wound was measured. Wound contraction was similar in the presence and absence of a rigid disc between the heart and the sternum during NPWT. Detailed results are shown in Figure 2.

### Wound distension under NPWT and traction

**Figure 3:**
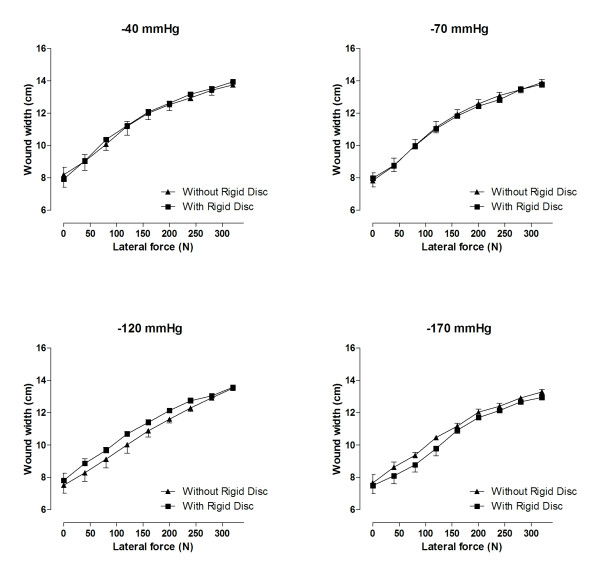
**Wound distension upon the application of a lateral force to draw apart the sternum wound edges to investigate the degree of stabilization during NPWT with and without a rigid barrier**. Negative pressures of -40, -70, -120 and -170 mmHg were applied, and the wound width was measured under traction forces between 0 and 320 N. It can be seen that the wound is stabilized to similar degrees in the absence and in the presence of a rigid barrier during NPWT.

After the application of each negative pressure, increasing levels of lateral traction were applied. This caused the sternum wound edges to be pulled apart. The increase in the width of the wound was determined at each force. The sternum wound distension upon traction was similar with and without the rigid disc during NPWT, indicating similar sternum stability. Different levels of negative pressure (-40, -70, -120 and -170 mmHg) allowed similar lateral distortion of the sternum wound edges. Detailed results are shown in Figure 3.

## Discussion

NPWT improves the healing of poststernotomy mediastinitis. One of the major advantages of applying NPWT to sternotomy wounds is that it stabilizes the sternum, which facilitates respiration and allows early mobilization [5,6]. However, complications associated with bleeding and heart rupture with lethal outcome have been reported in several studies [2,4,12]. The insertion of a rigid barrier between the heart and the sharp edges of the sternum has been suggested to prevent such complications [10]. In the present study, sternum wound contraction and stabilization in the presence and absence of a rigid barrier during NPWT were examined. These are important to ensure the safety and efficacy of the negative pressure treatment of a sternotomy wound.

### Wound contraction

Contraction is important in a sternotomy wound to both accelerate healing and stabilize the wound. Wound contraction was observed in when NPWT was applied, and was similar in the absence and presence of a rigid barrier disc. Wound contraction is known to result in mechanical deformation of the wound edge tissue [13-15], which results in shearing forces at the wound-dressing interface that will affect the cytoskeleton [16] and initiate a cascade of biological effects ultimately resulting in granulation tissue formation and wound healing [14]. Indeed, it has been shown that early changes in the size of a wound are correlated to the rate of wound healing [17].

### Sternum wound stabilization

A sternotomy wound requires certain safety measures with regard to exposed vital organs. The sternum wound edges move when the patient moves, coughs and breaths, and the sternum wound must be contracted and stabilized for the treatment to be considered safe. Sternum wound stabilization is also important to ensure adequate respiration and mobilization during NPWT [5,11]. In this study, sternum wound stabilization can be tested by applying a lateral traction force to pull the sternal edges apart and force the wound to open. The results show that even at low levels of negative pressure (-40 and -80 mmHg), the sternum is significantly stabilized. It has previously been reported that wound stabilization is similar at low levels of negative pressure (-50 to -100 mmHg) and high levels of negative pressure (-150 to -200 mmHg) [11]. The present study also shows that wound distension upon traction is similar in the absence and presence of a disc to protect the heart and lungs during NPWT. These results suggest that a rigid barrier can be safely placed in the sternotomy wound to protect the heart and lungs from damage and rupture during NPWT, with regard to sternum wound contraction and distension.

### Conclusions

The most feared complication of NPWT-treated poststernotomy mediastinitis is heart rupture. The cause of right ventricular rupture may be contact with the sharp sternal edges as the heart is drawn up towards the thoracic wall. The use of a rigid barrier between the heart and the edges of the sternum has been shown to prevent this movement, and has been proposed as a means of preventing heart rupture. In the present study we show that the sternum wound is contracted and stabilized equally well in the presence as in the absence of a rigid barrier disc, inserted between the heart and the sternal edges during NPWT. This study provides evidence that a rigid disc can safely be inserted over the heart, for protection during NPWT with regard to sternum wound contraction and stabilization.

## Competing interests

The authors declare that they have no competing interests.

## Authors' contributions

SL, RI & MM carried out the experimental studies. SL drafted the manuscript. MT participated in the sequence alignment. SL, RI & MM participated in the design of the study and performed the statistical analysis. All authors read and approved the final manuscript.
